# Within-person associations of reallocating recreational screen time to moderate-to-vigorous physical activity with depressive symptoms among university students with disabilities: an intensive longitudinal study

**DOI:** 10.3389/fpubh.2026.1813735

**Published:** 2026-07-31

**Authors:** Jixing Wu, Caizhu Gao, Mengchan Gao, Hang Yin

**Affiliations:** 1 School of Physical Education, Huaqiao University, Quanzhou, China; 2 School of Sports Science, Anshan Normal University, Anshan, China; 3 School of Physical Education, Changzhou Vocational Institute of Industry Technology, Changzhou, China

**Keywords:** accelerometry, distributional analysis, ecological momentary assessment, smartphone sensing, students with disabilities, within-person association

## Abstract

**Objective:**

College students with disabilities are at high risk of depression, and their daily life is dominated by excessive recreational screen time. Previous studies have relied on self-reported data and focused primarily on mean associations, potentially obscuring within-person heterogeneity. This study examined whether modeled reallocation of recreational screen time to moderate-to-vigorous physical activity was associated with daily PHQ-8 scores and whether the estimated within-person association varied across conditional quantiles of the daily PHQ-8 distribution.

**Methods:**

1,248 college students with disabilities were followed up for 12 months, combined with ecological momentary assessment. Recreational screen time and moderate-to-vigorous physical activity were objectively measured by mobile phone sensing and accelerometer, respectively. Fixed-effect isotemporal substitution model was used to analyze the average association, and fixed-effect quantile regression was used to examine the heterogeneity of the estimated association across conditional quantiles of daily PHQ-8 scores. This was an observational study; no exercise intervention was imposed.

**Results:**

Participants had an average of 317 min of daily recreational screen time and only 28 min of moderate-to-vigorous physical activity. A modeled 60-min reallocation of recreational screen time to moderate-to-vigorous physical activity was associated with a 1.65-point lower daily PHQ-8 score on average. The estimated association was weak and non-significant at the 10th conditional quantile and larger at the 90th conditional quantile. Exploratory subgroup estimates were numerically largest among students with mental disorders, high chronic pain, or high assistive technology dependence. Exploratory participant-level path analysis identified indirect statistical associations involving rumination and sense of accomplishment; because temporal ordering was unavailable, these estimates were not interpreted as mediated proportions or mechanisms.

**Conclusion:**

The estimated within-person association between modeled reallocation of recreational screen time to moderate-to-vigorous physical activity and daily PHQ-8 scores varied across the conditional outcome distribution, with larger negative coefficients at higher conditional quantiles. These observational findings are hypothesis-generating and should not be interpreted as causal effects, clinical benefits, or a basis for subgroup targeting or resource allocation.

## 1 Introduction

Disabled college students are a vulnerable group, who are facing unique physical and mental, social and environmental challenges, and are also experiencing a critical period of development and transformation (1). These challenges, including social exclusion, physical barriers, and ongoing psychosocial adaptation processes, are associated with their disproportionate burden of mental health (2). Epidemiological surveys have shown that the prevalence of depressive symptoms in this group is 1.5–2 times higher than that in the general student population (3). At the same time, a sedentary lifestyle characterized by high recreational screen time and inadequate physical activity of moderate to high intensity is prevalent among college students with disabilities, which may create a potential double burden, exacerbate psychological distress, and put a significant strain on both individual coping resources and institutional support systems (4). Existing research on the relationship between behavior and mental health in this context has limitations in two key aspects. Methodologically, the vast majority of studies rely on self-reported behavioral data (5, 6). Such reports are susceptible to recall error and social-desirability bias, potentially resulting in exposure misclassification (7). Conceptually, traditional regression methods mainly estimate mean associations. While these estimates are informative, they may mask heterogeneity across the conditional outcome distribution (8). These approaches therefore cannot determine whether the estimated within-person association of behavioral reallocation differs across positions in the daily depressive-symptom distribution. Characterizing this heterogeneity may generate hypotheses for future confirmatory and intervention studies (9).

Theoretical frameworks, such as conservation of resources (COR), provide a compelling perspective for predicting and interpreting heterogeneous associations (10, 11). COR theory holds that individuals strive to acquire, retain, and protect the resources they value. We hypothesized that at higher conditional quantiles of daily PHQ-8 scores, lower recreational screen time combined with higher moderate-to-vigorous physical activity might show a stronger negative association with daily PHQ-8 scores than at lower conditional quantiles (12, 13). This application of the principle of diminishing marginal utility provides a theoretical basis for expecting distributional heterogeneity (14). Empirical assessment of this pattern can generate hypotheses for future intervention studies rather than identify priority groups or expected benefits (15).

Methodological innovations have made more rigorous and detailed research possible. The combination of smartphone sensing technology with research-grade accelerometers allows for objective, continuous, and ecologically valid measurements of everyday behavior, effectively circumventing self-report bias (16). Individual fixed-effects models control for all participant characteristics that remain constant over time, but they do not eliminate time-varying confounding or reverse causality (17). When this approach is combined with fixed-effects quantile regression, it is possible to go beyond the mean and systematically map how behavioral associations vary across the entire conditional distribution of depressive symptoms (18). This distributional analysis is useful for describing heterogeneity that mean models may not capture.

Therefore, this study integrated device-based objective measures, a robust observational design, and distributional analysis to address the following questions: (1) Is device-measured reallocation of recreational screen time to moderate-to-vigorous physical activity associated with within-person differences in daily PHQ-8 scores among college students with disabilities? (2) Does the estimated association vary systematically across conditional quantiles of daily PHQ-8 scores? (3) Is this distributional pattern consistent across disability type subgroups or is it modified by disability-specific factors?

The category of “disability” encompasses a wide range of conditions that have varying impacts on physical activity participation. For example, students with physical disabilities may face mobility-related MVPA barriers, while those with sensory disabilities might encounter obstacles related to information or the environment. The “other” disability category is particularly heterogeneous, including rare or multiple diseases that hinder meaningful subgroup analysis. This diversity may affect the feasibility of behavioral reallocation and its association with depressive symptoms. Therefore, we consider disability types as key moderating variables in subgroup analysis, while acknowledging that the “other” category is retained solely for descriptive reference.

## 2 Methods

### 2.1 Study design and participants

This study used a dense longitudinal design that combines ecological momentary assessment with objective device-based monitoring (19). The total study period was 12 months, in which intensive data collection periods were conducted quarterly, each lasting 7 consecutive days. Participants were recruited from support service centers for students with disabilities at 10 universities in China through a stratified random sampling method. Research assistants contacted potential participants through the center’s mailing list and field visits. Interested individuals underwent eligibility screening, and those who met the criteria were provided with a detailed research information form. The stratification variables for random sampling were university, type of disability, and academic year. Inclusion criteria were: (1) possession of an official certificate of disability; (2) being a full-time registered college student; and (3) possession and daily use of a smartphone. Exclusion criteria were: (1) the presence of cognitive impairment so severe that the assessment could not be completed; (2) pregnancy or lactation; or (3) planned suspension from school during the study.

Among the initial 1,382 eligible students, 1,248 (90.3%) provided valid data for at least two assessments and were included in the final analysis sample. The completion rate of each wave was 1,248 (100% of the enrollment) for the first wave, 1,198 (96. 0%) for the second wave, 1,153 (92. 4%) for the third wave, and 1,104 (88.5%) for the fourth wave. The total number of valid participant days for all waves was 27,894 days (mean 22.4 days per participant, SD = 5.8). The accelerometer wear adherence rate was 89.7%, and the EMA completion rate was 86.2% (see Supplemental Figure 1 for participant flowcharts). Each wave required at least 4 valid days to be included. The final analytic sample consisted of 1,248 eligible college students with disabilities, with a mean age of 20.3 years (SD = 2.1), 56.3% of whom were male. Disability types are classified according to the official disability certificate and by reference to the framework of the International Classification of Functioning, Disability and Health (ICF) of the World Health Organization. The sample included 477 (38.2%) persons with physical disabilities, 501 (40.2%) persons with sensory impairments (including visual and hearing impairments), and 191 (15.3%) persons with mental disorders. An additional 79 participants (6.3%) were classified as having “other disabilities.” Given the high heterogeneity within the subgroup and the small sample size, it was retained in the primary analysis to ensure sample integrity, but was not interpreted substantively in the comparative analysis. The “Other” category includes participants with rare congenital diseases, multiple co-occurring disabilities, or conditions that cannot be classified into the three main domains of ICF; these participants are included in the main model but excluded from subgroup comparisons. Disability types were verified against official certificates by two trained researchers independently. The study protocol was approved by the Ethics Review Committee of the School of Sports Science, Anshan Normal University. Written informed consent to participate was obtained from all participants before enrollment.

### 2.2 Variables and measurement

#### 2.2.1 Device-measured time-use variables

It is worth noting that this is an observational measurement study. Participants were instructed to maintain their regular routines without any prescribed exercise plan. Therefore, the device-based measures described below reflect natural behavior rather than a response to structured interventions.

Recreational Screen Time: Objectively measured via smartphone sensing API (“screen time” for iOS and “digital health” for Android) combined with a self-developed monitoring app. Screen time data dedicated to entertainment purposes (e.g., social media, video streaming, gaming) is continuously recorded at 5-min intervals. The app uses a classification algorithm based on a predefined taxonomy: applications are categorized into entertainment (social media platforms, video streaming services, gaming applications, and entertainment websites) and education/productivity (learning management systems, office productivity suites, academic research tools, and language learning applications). For mixed-use applications (e.g., YouTube, Web browsers), the algorithm classifies usage based on primary activity inferred from time of day patterns (e.g., evening viewing is classified as entertainment) and usage duration, and marks ambiguous periods for review by the researchers. In the validation substudy (*n* = 120), participants completed daily activity diaries for 7 consecutive days; the algorithm achieved 91% agreement with diary entries (Cohen’s *κ* = 0.88, 95% CI: 0.82–0.94). Data from iOS and Android platforms were coordinated through standardized API calls to ensure comparability; we tested for platform differences in the primary analyses and found no significant interaction (*p* = 0.32 for platform × screen time interaction). Smartphone screen time can be objectively quantified using device-recorded usage logs (20). In the present study, applications were subsequently classified according to a study-defined rule set.

Moderate-to-Vigorous Physical Activity (MVPA): Objective assessment using an ActiGraph wGT3X-BT accelerometer worn on the right hip. Participants were asked to wear the device for at least 10 h per day while awake. The Freedson synthesis equation was used to calculate activity counts per minute, defining MVPA as activity with ≥2,690 counts per minute (21). The daily MVPA time is calculated by adding up the number of minutes that meet this criterion.

#### 2.2.2 Other time use components

Sleep duration: determined using accelerometer data in combination with sleep diary entries.

Educational Screen Time: Identified by Device Using Classification Algorithms.

Low-intensity physical activity: defined by activity with accelerometer counts in the range of 1,500–2,689 beats/min.

Other time: Remaining awake time is classified as containing activities such as eating, social interaction, and personal care.

#### 2.2.3 Outcome variables

Depressive symptoms: measured using the 8-item Patient Health Questionnaire (PHQ-8). The standard PHQ-8 has shown good reliability and validity in populations with disabilities. In this study, the PHQ-8 was administered daily through the EMA protocol at a fixed time each night and adapted to a 24-h reference frame (“over the past 24 h”) to capture day-to-day variation. This 24-h adaptation has not been formally validated against the standard 2-week PHQ-8 or clinical diagnostic interviews. Accordingly, daily PHQ-8 scores were analyzed primarily as continuous outcomes. Numerical score bands (1–4, 5–9, 10–14, and ≥15) are reported descriptively only and are not labeled or interpreted as clinical severity categories; standard diagnostic thresholds and minimum clinically important differences were not applied. The total score ranged from 0 to 24, with higher scores indicating more symptoms reported over the preceding 24 h (22).

#### 2.2.4 Time-varying covariates

Disability-related factors: daily pain intensity (0-10-point numerical rating scale), duration of assistive technology use (hours/day), and degree of limitation in daily activities (5-point Likert scale) (23).

Psychosocial factors: daily perceived stress (4-item short perceived stress scale), frequency and quality of face-to-face social interaction (5-point scale), and academic stress (3-item scale) (24).

Situational/environmental factors: course schedule (with or without courses), campus activity participation (yes/no), and weekends versus weekdays.

Other health behaviors: caffeine intake (cups/day) and psychotropic drug use (yes/no).

### 2.3 Data processing and quality control

The non-wearing time of the accelerometer is defined as a period of ≥60 consecutive minutes during which the count is zero. Sleep periods were identified using the Choi algorithm applied to the accelerometer data and validated with sleep diary entries. Data with active accelerometer wear time <600 min/day were excluded. Missing data (ranging from 2.1% for PHQ-8 to 8.7% for accelerometer wear time) were processed with multiple imputation to generate 20 complete data sets, and the results were combined using Rubin’s rule (25).

### 2.4 Statistical analysis

The analysis proceeded in three sequential stages. First, descriptive statistics characterized the sample. The intraclass correlation coefficient (ICC) for depressive symptoms was calculated. Second, fixed-effects isotemporal substitution models were employed to estimate the average within-individual association of time reallocation (26, 27). The model was specified as: Depression_it = *β*_0 + *β*_1*MVPA_it + *β*_2*Sleep_it + *β*_3*EducationalScreen_it + *β*_4*LPA_it + *β*_5*OtherTime_it + *γZ*_it + *α*_*i* + *ε*_it where *α*_*i* represents individual fixed effects, *Z*_it is a vector of time-varying covariates, and the coefficient *β*_1 represents the estimated change in depressive symptoms when reallocating 60 min from recreational screen time (the omitted reference component) to MVPA, holding all other time components constant. The isotemporal substitution model imposed the time-budget constraint that all time-use components (recreational screen, MVPA, sleep, educational screen, LPA, other time) sum to 1,440 min per day; recreational screen time was excluded from the model as the reference category. All models incorporated clustered standard errors at the participant level. Third, to unpack heterogeneity, fixed-effects quantile regression estimated this same association at five key conditional quantiles of the depressive symptom distribution (*τ* = 0.1, 0.25, 0.5, 0.75, 0.9) (28). Heterogeneity across quantiles was formally tested using Wald tests. All subgroup analyses were predefined in the study protocol; however, given the multiple comparisons and exploratory nature of interaction effects, these findings are considered hypothesis-generating, and results should be interpreted with caution. All analyses were conducted using Stata 18.0 and R 4.2.0. Confidence intervals for the quantile regression coefficients were obtained from 1,000 bootstrap replications with participant-level resampling (i.e., cluster bootstrapping) to preserve the within-person correlation structure. Significance was set at *α* = 0.05 (two-sided).

Chained equations (MICE) were used for multiple imputation of 20 imputation data sets, including all variables in the analysis model as predictors. The imputation model included outcomes (PHQ-8), all time use components (MVPA, sleep, educational screening, LPA, other time), and all time-varying covariates. The results are combined using Rubin’s rule (29). Sensitivity analyses included: (a) adjustment for BMI change, but not baseline BMI; (B) exclusion of accelerometer wear day outliers (mean > 2 SD); (c) additional adjustment for sleep quality; (d) exclusion of the “other disability” grouping; and (e) use of the full case analysis as an alternative to multiple imputation (30).

For exploratory participant-level path and subgroup analyses, the following considerations apply. First, the path analysis used participant-level averages of all available waves rather than temporally ordered repeated measures; therefore, it describes cross-sectional statistical associations and does not establish temporal priority, indirect causal effects, or mechanisms. Second, because the data were pooled to the participant level for the path analysis, repeated-measure dependencies were not an issue; for subgroup analyses, participant-level clustered standard errors were used in all quantile regression models to account for repeated observations. Third, the subgroup with daily PHQ-8 scores ≥15 and the two intermediate variables (rumination and accomplishment) were prespecified in the study protocol; all subgroup analyses were also prespecified. Fourth, given the exploratory and hypothesis-generating nature of these analyses, no adjustments were made for multiple comparisons (e.g., Bonferroni correction). We acknowledge that this increases the risk of Type I error; therefore, these findings should be interpreted with caution and confirmed in future studies. All subgroup and path-analysis results are clearly labeled “Exploratory” in the Results section.

## 3 Results

### 3.1 Sample characteristics and behavioral patterns

The sample for analysis included 1,248 college students with disabilities (56.3% male, mean age = 20.3 ± 2.1 years). The types of disability included physical disability (38.2%), sensory impairment (40.2%), mental disorder (15.3%) and other disability (6.3%). As shown in Table 1, significant differences were observed across disability groups for age, socioeconomic status, and disability-related characteristics (all *p* < 0.05).

**Table 1:** Sample baseline characteristics and device-measured behaviors (*N* = 1,248).

Characteristic category	Variable	Total sample (*N* = 1,248)	Physical disability (*n* = 477)	Sensory impairment (*n* = 501)	Mental disorder (*n* = 191)	Other disabilities (*n* = 79)	*p*-value
Demographics	Age (years)	20.3 ± 2.1	21.2 ± 2.3	19.8 ± 1.9	19.5 ± 1.8	20.6 ± 2.2	<0.001
	Sex (male)	703 (56.3%)	285 (59.7%)	292 (58.3%)	89 (46.6%)	37 (46.8%)	0.003
	Years in University	2.4 ± 1.3	2.6 ± 1.4	2.3 ± 1.2	2.1 ± 1.1	2.5 ± 1.3	0.002
Socioeconomic status	Household monthly income (¥10,000)	1.2 ± 0.8	1.1 ± 0.7	1.3 ± 0.9	1.0 ± 0.6	1.2 ± 0.8	0.001
	Receiving financial aid	843 (67.5%)	335 (70.2%)	327 (65.3%)	142 (74.3%)	39 (49.4%)	<0.001
	Part-time job	287 (23.0%)	98 (20.5%)	125 (24.9%)	42 (22.0%)	22 (27.8%)	0.352
Disability-related	Years since disability onset	8.7 ± 6.2	10.3 ± 7.1	7.2 ± 5.3	6.8 ± 4.9	9.1 ± 6.5	<0.001
	Multiple disabilities	156 (12.5%)	45 (9.4%)	62 (12.4%)	38 (19.9%)	11 (13.9%)	0.005
	Limitations in daily activities (1–5)	3.2 ± 1.1	3.8 ± 0.9	2.7 ± 1.0	3.1 ± 1.2	3.4 ± 1.1	<0.001
Device-measured behaviors (min/day)	Recreational screen time	317 ± 142	285 ± 135	325 ± 148	389 ± 139	298 ± 132	<0.001
	Moderate-to-vigorous physical activity (MVPA)	28 ± 19	22 ± 16	31 ± 20	25 ± 17	30 ± 19	<0.001
	Sleep time	458 ± 73	462 ± 71	455 ± 75	443 ± 74	461 ± 70	0.023
	Educational screen time	135 ± 68	128 ± 65	142 ± 70	131 ± 67	129 ± 66	0.018
	Light-intensity physical activity (LPA)	195 ± 84	183 ± 81	201 ± 86	192 ± 83	199 ± 82	0.007
Mental health indicators	PHQ-8 total score	7.8 ± 5.2	6.9 ± 4.8	7.5 ± 5.1	10.3 ± 5.7	7.2 ± 4.9	<0.001
	Daily PHQ-8 score (1–4)	312 (25.0%)	138 (28.9%)	132 (26.3%)	25 (13.1%)	17 (21.5%)	<0.001
	Daily PHQ-8 score (5–9)	523 (41.9%)	205 (43.0%)	213 (42.5%)	68 (35.6%)	37 (46.8%)	
	Daily PHQ-8 score (10–14)	287 (23.0%)	98 (20.5%)	115 (23.0%)	57 (29.8%)	17 (21.5%)	
	Daily PHQ-8 score (15+)	126 (10.1%)	36 (7.5%)	41 (8.2%)	41 (21.5%)	8 (10.1%)	
Other health indicators	Chronic pain intensity (0–10)	4.2 ± 2.8	5.8 ± 2.5	3.1 ± 2.6	4.5 ± 2.9	4.0 ± 2.7	<0.001
	Assistive technology use (hours/day)	3.8 ± 2.5	4.2 ± 2.6	3.9 ± 2.4	2.8 ± 2.3	4.1 ± 2.7	<0.001
	Perceived stress (4–20)	12.7 ± 3.9	12.3 ± 3.8	12.5 ± 3.9	14.2 ± 4.1	12.1 ± 3.7	<0.001

Continuous variables are presented as Mean ± Standard Deviation; categorical variables are presented as Frequency (Percentage). *p*-values were derived from one-way ANOVA (for continuous variables) or chi-square tests (for categorical variables) to compare differences across disability type groups. The “Sensory Impairment” group includes visual and hearing impairments. Due to high internal heterogeneity, the descriptive statistics for the “Other Disabilities” subgroup are presented for reference only and should not be used for primary comparative interpretation.

**Figure 1:**
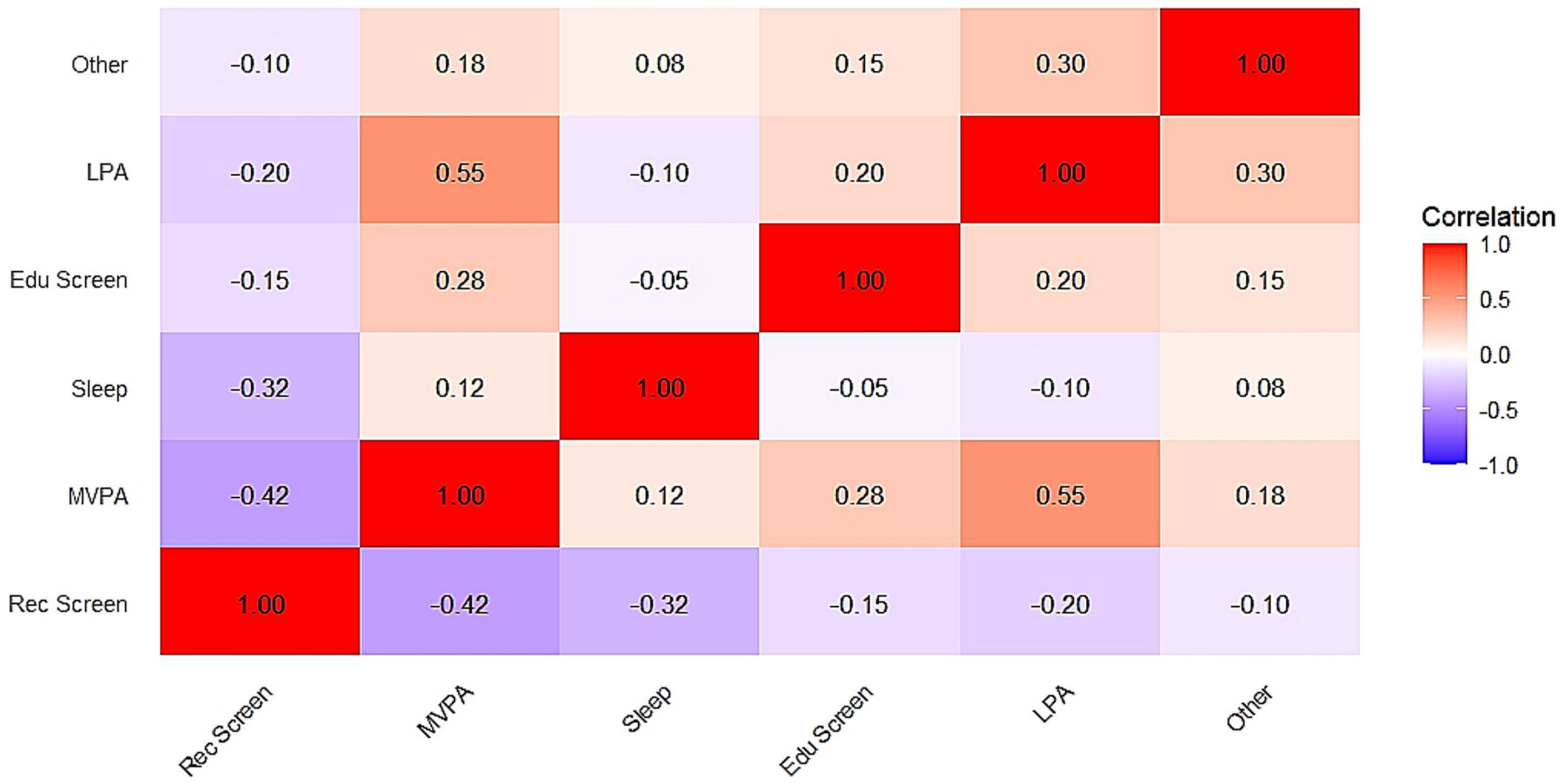
Correlation matrix of daily time-use components. The value is the Pearson correlation coefficient (*N* = 1,248). Blue indicates negative correlation, while red indicates positive correlation. All principal components showed significant interrelationships, with entertainment screen time being negatively correlated with MVPA and sleep.

Device-based objective measures showed significant differences in daily time use patterns (Table 1). Participants spent an average of 317 ± 142 min of daily recreational screen time, compared with only 28 ± 19 min of moderate-to-vigorous physical activity, well below public health recommendations. Correlation analysis (Figure 1) indicated that there was a significant correlation between the components of time use: recreational screen time was moderately negatively correlated with both MVPA (*R* = −0.42) and sleep (*R* = −0.32), while MVPA was positively correlated with low-intensity physical activity (*R* = 0.55).

**Figure 2:**
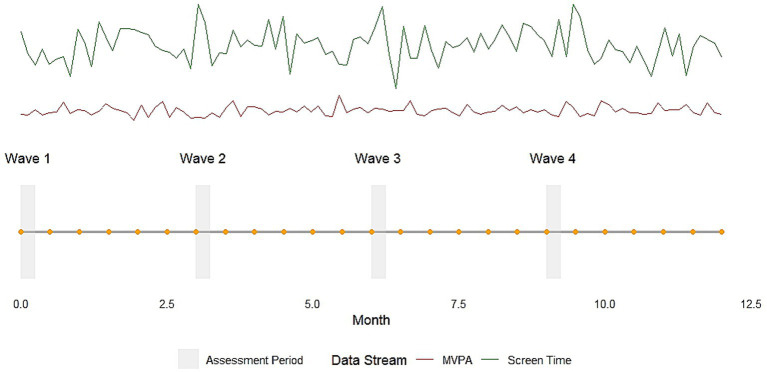
Schematic of the 12-month intensive longitudinal study design. Data collection is conducted over four quarters, with each quarter lasting 7 days. It integrates three data streams: entertainment screen time derived from smartphones (green), MVPA measured by accelerometers (red), and PHQ-8 scores assessed daily via EMA (orange dots).

The mean daily PHQ-8 score was 7.8 ± 5.2, with significant differences across disability groups. Students with mental disorders reported the highest mean daily score (10.3 ± 5.7), and 21.5% had daily PHQ-8 scores ≥15. This numerical score band is descriptive and is not a clinical severity category. The intra-class correlation coefficient for daily PHQ-8 scores was 0.41, indicating significant intra-individual variation (59%), which justifies the use of an intra-individual model. This within-individual variation was captured by our intensive longitudinal design (Figure 2), which combined ecological momentary assessments with continuous device-based monitoring over a 12-month period, supporting subsequent robust within-individual analyses.

### 3.2 Average within-person association of behavioral reallocation

**Figure 3:**
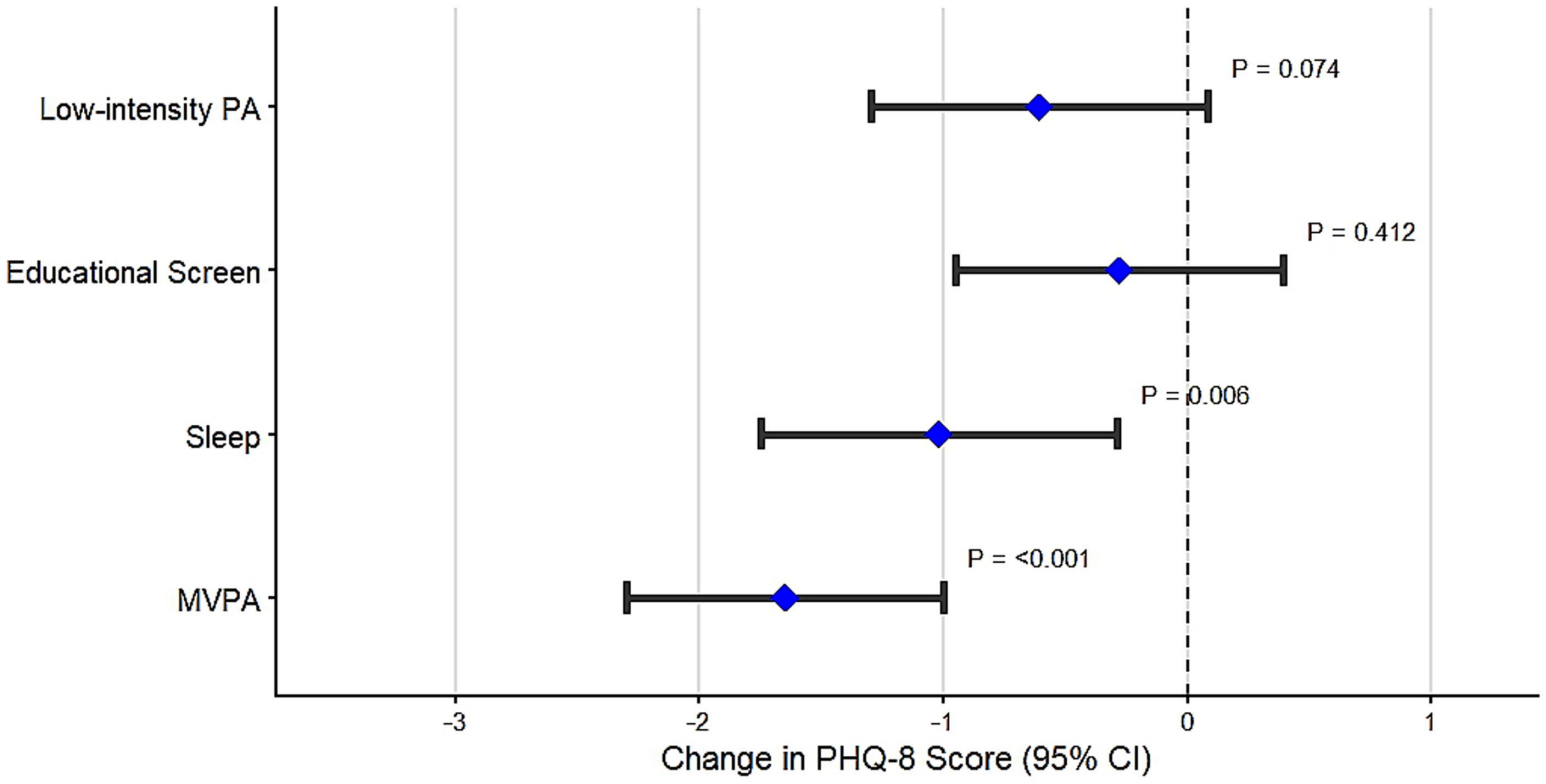
Fixed-effects isotemporal substitution estimates for reallocating 60 min of screen time to other behaviors. Points and error bars represent the *β* coefficient and 95% CI (*N* = 1,248). Modeled reallocation to MVPA and sleep was significantly associated with lower daily PHQ-8 scores, whereas the estimated associations for educational screen time and LPA were not statistically significant.

The fixed-effects isotemporal substitution model revealed a significant mean within-person association between behavioral reallocation and daily PHQ-8 scores (Figure 3). A modeled reallocation of 60 min of device-measured recreational screen time to device-measured MVPA was associated with an estimated −1.65-point within-person difference in daily PHQ-8 score (95% CI, −2.30, −1.00; *p* < 0.001). The normalized coefficient was −0.32. No clinical-importance threshold was applied to the 24-h adapted PHQ-8.

Comparative analysis showed that modeled reallocation of recreational screen time to sleep was also associated with a lower daily PHQ-8 score (*β* = −1.02; 95% CI: −1.75, −0.29; *p* = 0.006), whereas the estimated associations for educational screen time (*β* = −0.28; *p* = 0.412) and low-intensity physical activity (*β* = −0.61; *p* = 0.074) were not statistically significant. Sensitivity analyses produced similar association estimates across model specifications, ranging from −1.49 to −1.71 for the screen time → MVPA contrast.

### 3.3 Gradient of associations across conditional quantiles

**Figure 4:**
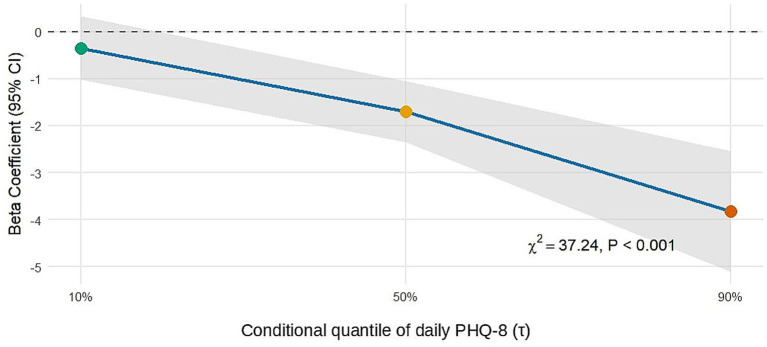
Quantile regression estimates across conditional quantiles of daily PHQ-8 scores. The estimated within-person association became more negative from the 10th to the 90th conditional quantile (*N* = 1,248). Conditional quantiles are positions in the outcome distribution and are not clinical severity groups. Coefficients differed across quantiles (Wald test *χ*^2^ = 37.24, *p* < 0.001).

The average within-person association masked heterogeneity across the conditional distribution of daily PHQ-8 scores (Figure 4). Quantiles represent positions in the conditional outcome distribution and are not baseline symptom groups or clinical categories. At the 10th conditional quantile (*τ* = 0.1), the estimated association was weak and not statistically significant (*β* = −0.35; 95% CI: −1.02, 0.32; *p* = 0.304). The coefficient was −1.70 at the median (*τ* = 0.5; 95% CI: −2.35, −1.05; *p* < 0.001) and −3.82 at the 90th conditional quantile (τ = 0.9; 95% CI: −5.10, −2.54; *p* < 0.001). The estimated coefficients differed across quantiles (Wald test for quantile heterogeneity: *χ*^2^ = 37.24, *p* < 0.001).

### 3.4 Exploratory heterogeneity across key subgroups

**Figure 5:**
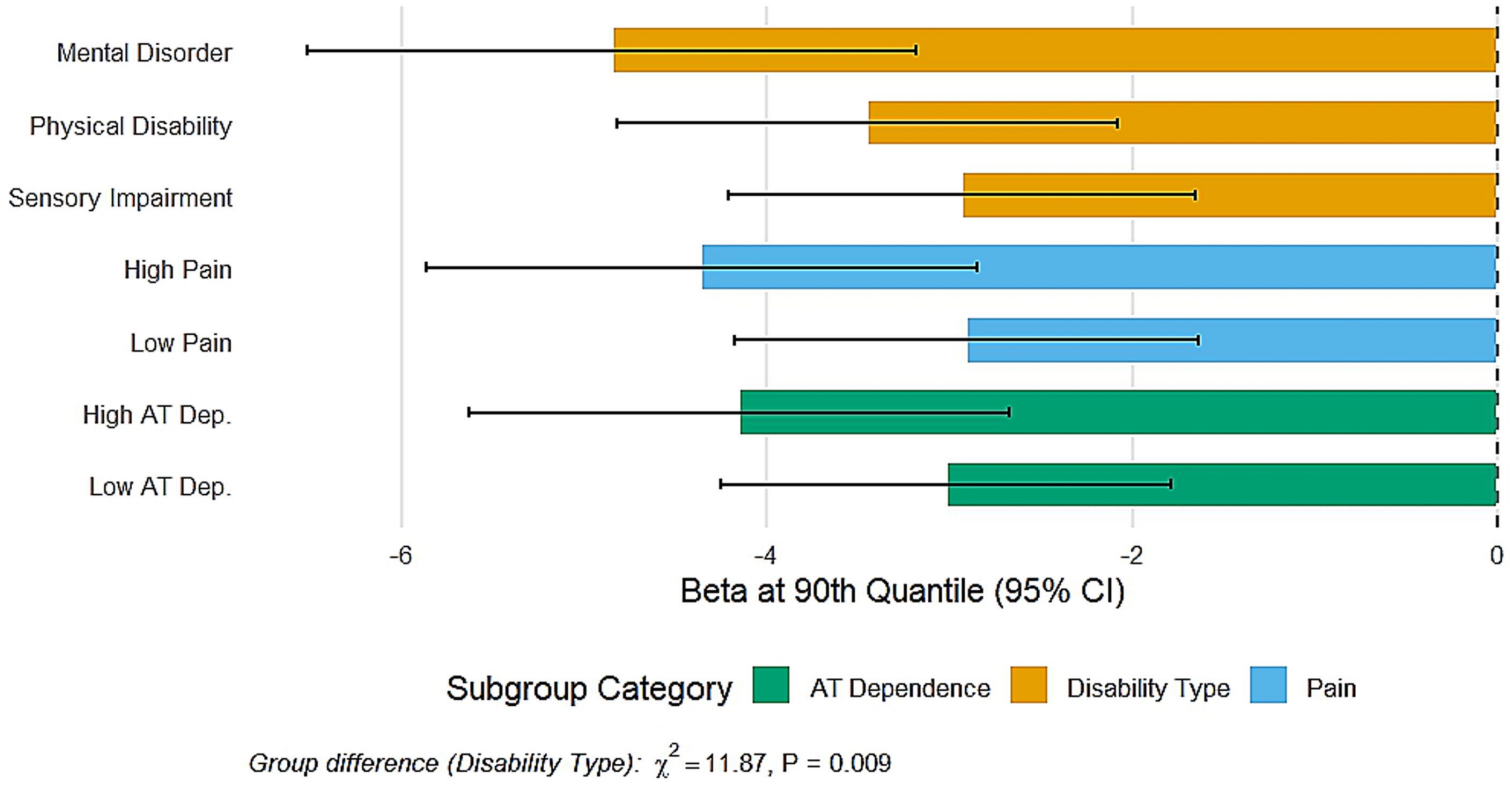
Exploratory subgroup-specific estimated associations at the 90th conditional quantile. Points and error bars show *β* coefficients and 95% confidence intervals stratified by disability type, pain level, assistive technology (AT) dependence, and social support. Differences across disability types were statistically significant (*χ*^2^ = 11.87, *p* = 0.009). These unadjusted multiple subgroup comparisons are hypothesis-generating.

Exploratory subgroup analysis showed heterogeneity in the estimated association at the 90th conditional quantile across demographic and disability-related characteristics (Figure 5). The largest negative coefficient occurred in the mental disorder subgroup (τ = 0.9: *β* = −4.85; 95% CI: −6.52, −3.18), followed by the physical disability subgroup (*β* = −3.45; 95% CI: −4.82, −2.08) and the sensory impairment subgroup (*β* = −2.93; 95% CI: −4.21, −1.65). The difference across disability groups was statistically significant (*χ*^2^ = 11.87, *p* = 0.009). Because no adjustment for multiple comparisons was applied, these subgroup estimates are hypothesis-generating.

Chronic pain intensity and assistive technology dependence were also associated with heterogeneity at the 90th conditional quantile. Compared with those with low pain levels (*β* = −2.91; 95% CI: −4.18, −1.64), those with high pain levels had a larger negative coefficient (*β* = −4.36; 95% CI: −5.87, −2.85; *p* value for difference = 0.018). Similarly, high assistive technology dependence (≥4 h/day) was associated with a larger negative coefficient (*β* = −4.15; 95% CI: −5.63, −2.67; *p* = 0.026) than low dependence (*β* = −3.02; 95% CI: −4.25, −1.79). Social-support groups also differed, with a smaller negative coefficient in the high-support group.

### 3.5 Exploratory participant-level path association analysis in the higher daily PHQ-8 score subsample (daily PHQ-8 ≥15)

**Figure 6:**
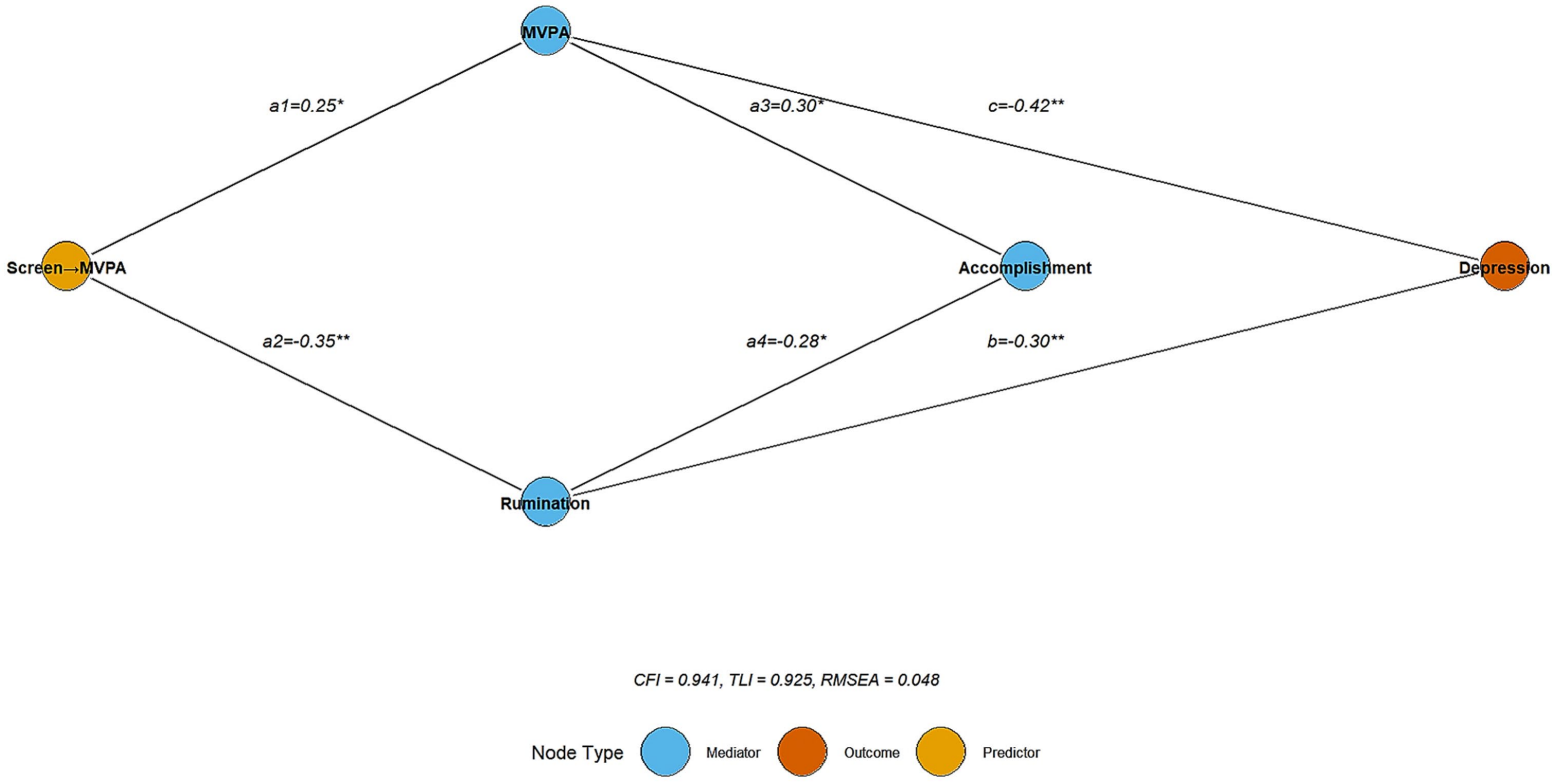
Exploratory participant-level path association model in the subgroup with daily PHQ-8 scores ≥15 (*n* = 126). Standardized coefficients are shown for cross-sectional associations among participant-level averages. Product-of-coefficients estimates involving rumination and accomplishment are reported without percentages mediated. Because temporal ordering was unavailable, the model does not establish mediation or mechanisms. Model fit: CFI = 0.941, TLI = 0.925, RMSEA = 0.048.

Exploratory participant-level path analysis in the subgroup with daily PHQ-8 scores ≥15 (*n* = 126) identified two indirect statistical associations (Figure 6). The overall association between the screen time → MVPA contrast and daily PHQ-8 score was −3.82 (95% CI: −5.10, −2.54). The product-of-coefficients estimate for the path involving nightly rumination was *β* = −0.68 (95% CI: −1.12, −0.24), and the corresponding estimate for the path involving daily accomplishment was *β* = −0.72 (95% CI: −1.18, −0.26). The model showed acceptable fit (CFI = 0.941, TLI = 0.925, RMSEA = 0.048). Because all variables were averaged across waves and temporal ordering was unavailable, percentages mediated were not calculated or interpreted, and these estimates do not establish mediation or mechanisms.

### 3.6 Individual symptom-behavior co-variation trajectories

**Figure 7:**
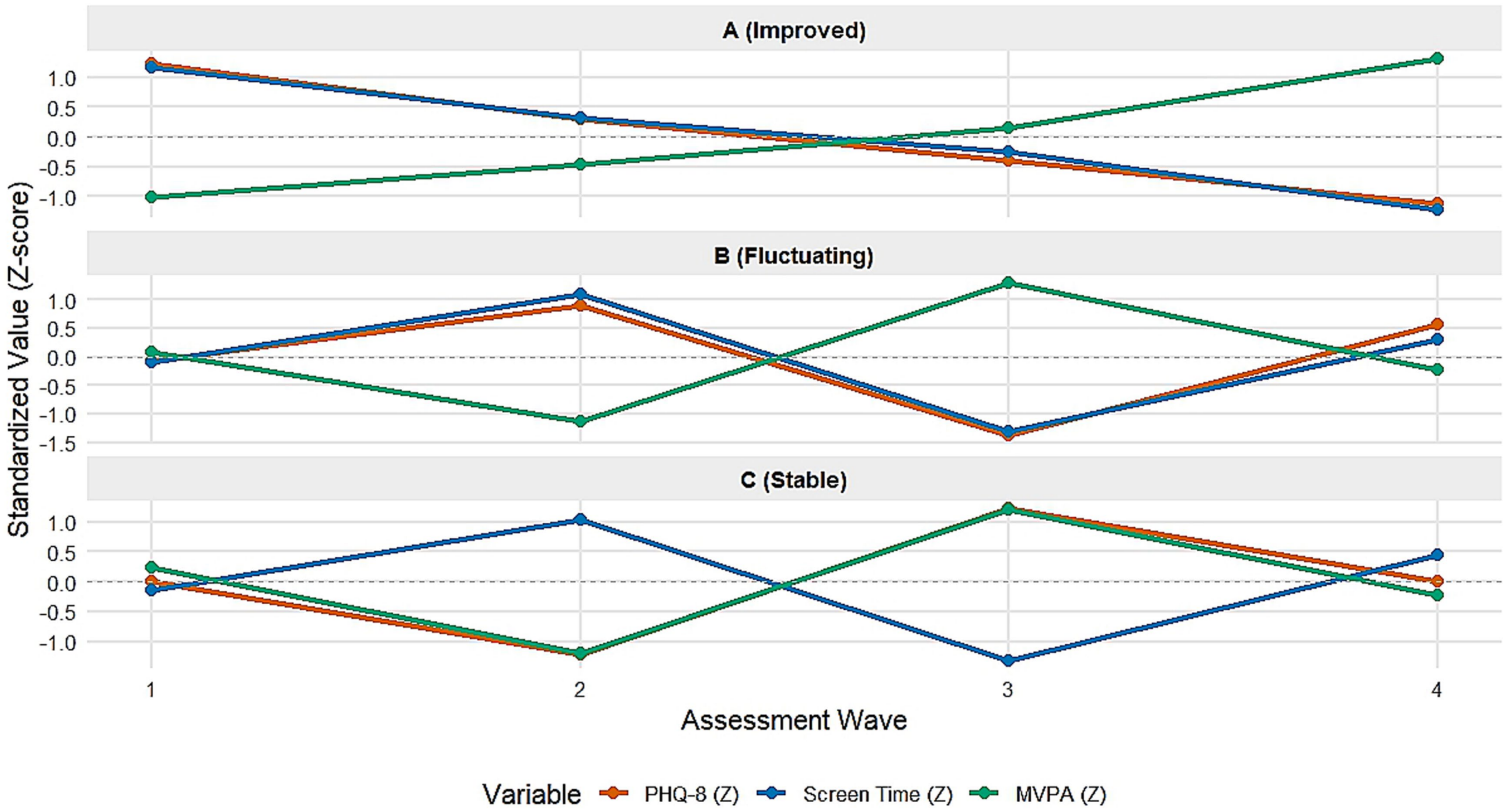
Intra-individual co-variation trajectories for three representative cases. Each panel shows standardized Z scores for PHQ-8, recreational screen time, and MVPA across four waves. Case A shows synchronous improvement; Case B exhibits fluctuating parallel changes; Case C maintains a stable pattern.

Individual-level trajectory analysis illustrates co-variation between behavioral patterns and daily PHQ-8 scores (Figure 7). Three representative cases show different descriptive patterns: Case A (“decreasing PHQ-8”) showed concurrent decreases in recreational screen time and daily PHQ-8 scores and an increase in MVPA across the assessment period. Case B (“fluctuating”) showed synchronous fluctuations in screen time, MVPA, and daily PHQ-8 scores. Case C (“relatively stable”) maintained comparatively stable behavioral and score patterns. These trajectories illustrate within-person co-variation and do not establish temporal or causal direction.

### 3.7 Exploratory subgroup pattern visualization for hypothesis generation

**Figure 8:**
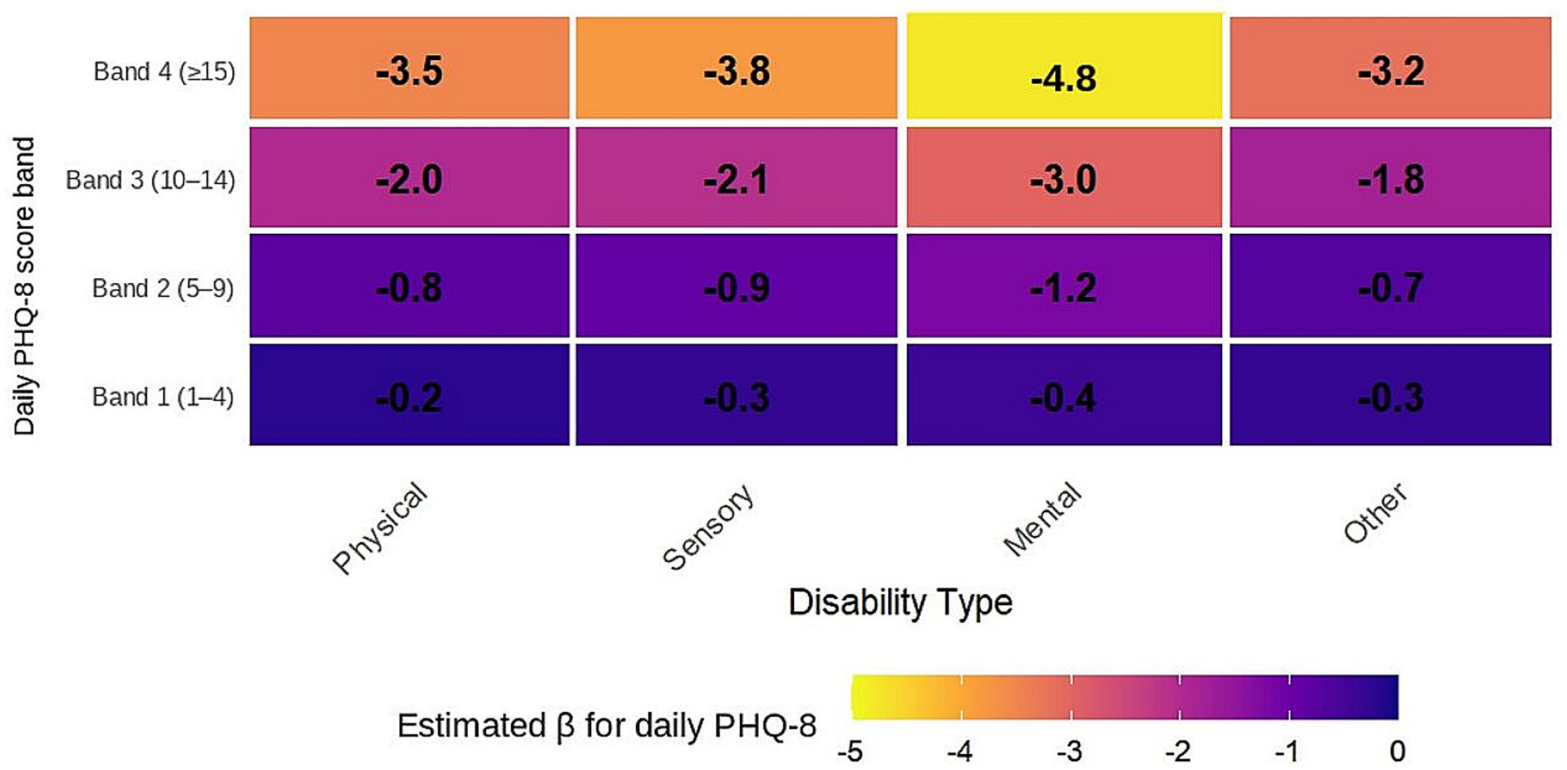
Exploratory association matrix by descriptive daily PHQ-8 score band and disability type. Cell values represent estimated *β* coefficients for combinations of descriptive daily PHQ-8 score bands and disability type (*N* = 1,248). Score bands are not clinical categories, and the visualization is intended only for hypothesis generation; it does not identify priority groups or guide resource allocation.

To summarize exploratory heterogeneity, Figure 8 presents estimated associations by descriptive daily PHQ-8 score band and disability type. The numerical score bands are not clinical categories. This visualization is descriptive and hypothesis-generating; it does not rank subgroups or support behavioral targeting.

The largest numerical estimate occurred in the daily PHQ-8 score band ≥15 among participants with mental disorders (*β* ≈ −4.85). Given the observational design, the use of a 24-h adapted PHQ-8, and the absence of multiple-comparison correction, differences in these estimates should not be interpreted as clinical benefits or used to allocate resources. They identify combinations of characteristics for confirmatory evaluation in future preregistered intervention studies.

### 3.8 Sensitivity and robustness analyses

All major findings remained robust in the multiple sensitivity analysis. Replacing baseline BMI with BMI change as a covariate did not substantially change the effect estimates. The results were consistent after excluding abnormal values or adjusting sleep quality. Excluding the heterogeneous “other disability” subgroup did not alter the pattern of findings. Multiple imputation for missing data yielded results comparable to the full case analysis, supporting the robustness of our conclusions to potential missing data bias.

## 4 Discussion

This study is the first to systematically assess the distributional association between device-measured recreational screen time reallocation to moderate-to-vigorous physical activity and depressive symptoms among college students with disabilities. The core findings suggest significant and quantifiable heterogeneity in this association: The core findings suggest significant and quantifiable heterogeneity in this association: stronger inverse associations were observed at higher conditional quantiles of daily PHQ-8 scores, whereas comparatively smaller associations were observed at lower conditional quantiles. This pattern is largely consistent with our assumptions from the conservation of resources theory. The pattern remained consistent across disability subgroups, but was particularly evident in individuals with psychiatric disorders, with chronic pain, and with a high dependency on assistive technology.

### 4.1 Theoretical implications and interpretation

The results support and extend the application of resource conservation theory to the disabled population. The observed gradient association, that is, the association strength among individuals with higher depressive symptom levels is substantially larger than that of individuals with lower symptom levels, provides quantitative evidence for hypothesis generation regarding future intervention studies. This pattern may be interpreted in light of the core principle of COR theory: individual efforts to maintain, protect, and acquire resources. For individuals with higher depressive symptoms, whose psychological and physical resources may be depleted, reallocating time away from passive and potentially resource-consuming behaviors, such as recreational screen time, which may exacerbate social comparisons (31) and sleep disorders (32), toward resource-generating behaviors may be particularly relevant. For example, moderate-to-high-intensity physical activity, which cultivates a sense of mastery (33), provides physiological rewards, and provides social opportunities (34), may have a synergistic resource replenishment effect. The significantly greater associations observed in this population suggest that behavioral reallocation may correspond to critical nodes of resource deficits, consistent with the principle of diminishing marginal utility as applied to mental resources (35). This reverse application of the principle of diminishing marginal utility, in which initial resource acquisition yields the greatest marginal gain for the least resourceful individual, provides a possible economic rationale for the gradient associations we observe.

The differences in the strength of association between different types of disability further clarify the importance of interaction between people and the environment. The strongest association was observed among students with mental disorders, possibly reflecting the group’s higher emotional responsiveness to screen content (36) and greater sensitivity to the emotional regulatory effects of physical activity (37). Specifically, individuals with mental disorders may be more susceptible to the negative emotional impact of passive and social comparison-laden screen content, which may be related to neurophysiological pathways proposed to influence depressive symptoms (34). This may be related to specific psychopathological mechanisms underlying certain mental disorders, such as the role of umination in depression or anxiety, which may be related to passive media consumption (38) and may also be influenced by cognitive distraction and physiological arousal associated with MVPA (39). In contrast, relatively weak gradients in the sensory impairment subgroup suggest that disability-specific factors may produce association modification. For example, people with visual impairment may benefit more from auditory-guided physical activities such as guided running or goalball (40), while people with hearing impairment may respond better to visually cued activities (41). Our current aggregated classification may mask these subtle needs and will need to be more finely differentiated in future studies.

Chronic pain and high assistive technology dependence, as proxies for greater functional limitations, may exacerbate the vicious cycle of sedentary behavior and psychological distress by limiting activity choices and increasing costs associated with environmental adaptation (42). Therefore, reallocating time toward MVPA may be associated with partial interruption of this cycle by broadening behavioral choices and enhancing perceived control, resulting in greater observed associations (43).

### 4.2 Methodological contributions

This study advances the methodological frontiers of health behavior research by integrating device-based objective measures, a rigorous observational design, and distributional analysis. Device data provide a more precise and less biased measure of behavior than traditional self-report, which is particularly critical in populations with disabilities where cognitive differences may affect recall (44). Fixed-effects isotemporal substitution models effectively control for all confounding factors that do not change over time, such as genetic factors or stable personality traits, by using intra-individual variation. In addition, quantile regression goes beyond the limitation of estimating only the average association and reveals the differential association of behavioral redistribution among subgroups defined by needs, thus providing a useful framework for examining heterogeneity in behavioral associations and informing future methodological applications in public health research (45).

### 4.3 Practical and policy implications and feasibility considerations

Feasibility must be a central consideration when translating research findings into practice. Although this study highlights the potential relevance of behavioral redistribution, its practical implementation must be carefully evaluated. Baseline data show that the overall level of moderate-to-vigorous physical activity in this population, 28 min per day, is well below public health recommendations (46), and the complete reallocation of 60 min of screen time to moderate-to-vigorous physical activity is a considerable challenge. Importantly, although a 60-min reallocation is the analytical unit for this component analysis, given that the baseline MVPA is only 28 min per day, directly translating this into a uniform intervention target may be unrealistic for many people. Instead, our findings emphasize that even smaller incremental reallocations for individuals at higher conditional quantiles of daily PHQ-8 scores may be associated with potentially meaningful differences in depressive symptom scores. Therefore, our results should be interpreted as estimates of association under the modeled reallocation scenario rather than immediate intervention targets. They provide quantitative evidence for identifying subgroups with stronger observed associations and for estimating potential associations.

In practice, future intervention studies may consider evaluating incremental and individualized strategies. Consistent with the aforementioned heterogeneity, MVPA levels vary significantly across different types of disabilities (Table 1), ranging from 22 min per day for physical disabilities to 31 min per day for sensory impairments. This variation underscores the need to develop tailored physical activity plans for people with disabilities rather than a one-size-fits-all approach. For example, starting with a smaller reallocation, such as 15–20 min per day, combined with a tailored adaptive physical activity program, such as wheelchair basketball or blind guided athletics, and comprehensive accessibility support, can reduce barriers to participation and ensure the accessibility and sustainability of the intervention (47).

The findings have potential implications for campus mental health and disability support services, pointing to a multi-component and individualized support strategy. First, at the level of individual support, university disability centers could consider incorporating behavioral pattern monitoring into routine mental health screening. Students identified as having the triad of high screen time, low physical activity, and high depressive symptoms constitute a subgroup with stronger observed associations, and future studies should evaluate whether timely individualized counseling based on the principles of behavioral activation theory may be beneficial (48). To effectively translate this recognition into action, future implementation research could examine how institutional resources might be directed to program development and environmental modification. This could include evaluating the allocation of campus health promotion funding to the creation of structured and adaptive moderate-to-vigorous physical activity programs for different disabilities (49), while ensuring universal accessibility of sports facilities and reducing environmental barriers to participation (50). Finally, in order to provide real-time and scalable support, our findings may inform the future development and evaluation of immediate adaptive intervention systems. Such digital health tools could potentially leverage the same objective behavioral data used in this study to automatically provide contextualized and feasible microactivity recommendations when risks are detected, such as prolonged recreational screen use, especially for individuals with high depressive symptoms (51).

### 4.4 Limitations and future directions

There are some limitations in this study. First, although we controlled for a wide range of time-varying confounding factors, we cannot completely rule out the possibility of residual confounding, which is an inherent limitation of observational studies. Second, reverse causality cannot be entirely excluded; individuals with more severe depressive symptoms may inherently have more screen time and less MVPA. While our fixed-effects approach controlled for time-invariant factors, the possibility of time-varying confounding factors (such as acute stressors or medication changes) still remains. Third, the use of daily PHQ-8 with a 24-h reference frame, while appropriate for EMA, has not been formally validated against clinical diagnostic interviews. Therefore, our PHQ-8 score groups are used descriptively rather than diagnostically. Device measures, while objective, are unable to capture the content and psychological context of screen use; future research should incorporate content analysis to deepen understanding of the mechanisms. While the sample covered the major disability types, some low prevalence disability subgroups had limited representation. In addition, exploratory analyses in the high-symptom subgroup suggested possible associations with reduced rumination and enhanced sense of accomplishment; however, because these analyses were based on participant-level averages without temporal ordering, they should not be interpreted as evidence of mediation. Nonetheless, they provide initial hypothesis-generating insights. Future research should prioritize longitudinal experiments or quasi-experimental designs to test the observed heterogeneous associations, explore the long-term persistence of these associations, and develop multi-level intervention strategies that combine individual-level approaches and environmental modifications.

## 5 Conclusion

In summary, reallocation of recreational screen time to moderate-to-vigorous physical activity was associated with lower daily PHQ-8 scores within individuals, and the estimated associations varied between conditional quantiles of the daily PHQ-8 distribution. Exploratory subgroup and participant-level path estimates generated additional hypotheses, but they did not establish causal relationships, clinical benefits, or mediating mechanisms, nor did they provide a basis for subgroup targeting or resource allocation. Before practical recommendations can be made, confirmatory intervention studies using validated clinical outcomes and chronological measures are needed.

## Data Availability

The raw data supporting the conclusions of this article will be made available by the authors, without undue reservation.

## Supplementary material

SUPPLEMENTARY FIGURE 1 Participant flow chart. Of the 1,382 eligible students initially contacted, 1,248 (90.3%) provided valid data on at least two assessment waves and were included in the final analysis sample. Completion rates by wave and the number of observations included in each analysis are shown. The accelerometer wear adherence was 89.7%, and the EMA completion rate was 86.2%.

## References

[1] Lipka O Sarid M Aharoni Zorach I Bufman A Hagag AA Peretz H. Adjustment to higher education: a comparison of students with and without disabilities. Front Psychol. (2020) 11:923. doi: 10.3389/fpsyg.2020.00923, 32670127 PMC7332748

[2] Myers A Halpern-Manners A McLeod JD. Invisible disabilities and college academic success: new evidence from a mediation analysis. Soc Sci Res. (2024) 123:103058. doi: 10.1016/j.ssresearch.2024.103058, 39256022 PMC11399884

[3] Li L Wang P Zhao Q Liu Z Li S Wang X. Latent profile analysis of depressive symptoms in college students and its relationship with physical activity. J Affect Disord. (2024) 351:364–71. doi: 10.1016/j.jad.2024.01.214, 38296059

[4] Deyo A Wallace J Kidwell KM. Screen time and mental health in college students: time in nature as a protective factor. J Am Coll Heal. (2024) 72:3025–32. doi: 10.1080/07448481.2022.2151843, 36796079

[5] Tang S Werner-Seidler A Torok M Mackinnon AJ Christensen H. The relationship between screen time and mental health in young people: a systematic review of longitudinal studies. Clin Psychol Rev. (2021) 86:102021. doi: 10.1016/j.cpr.2021.102021, 33798997

[6] Brown DMY Faulkner GEJ Kwan MYW. Healthier movement behavior profiles are associated with higher psychological wellbeing among emerging adults attending post-secondary education. J Affect Disord. (2022) 319:511–7. doi: 10.1016/j.jad.2022.09.111, 36162673

[7] Lee PH Tse ACY Wu CST Mak YW Lee U. Validation of self-reported smartphone usage against objectively-measured smartphone usage in Hong Kong Chinese adolescents and young adults. Psychiatry Investig. (2021) 18:95–100. doi: 10.30773/pi.2020.0197, 33517618 PMC7960745

[8] Ni RJ Yu Y. Relationship between physical activity and risk of depression in a married group. BMC Public Health. (2024) 24:829. doi: 10.1186/s12889-024-18339-7, 38491473 PMC10943876

[9] Li P Alhumaid MM Wang H Li H Zhao S. Correlation research on physical activity and executive function in female college students with subclinical depression. Front Psych. (2024) 15:1403471. doi: 10.3389/fpsyt.2024.1403471, 38835550 PMC11148427

[10] Baumunk MJ Tang X Rumrill SP Conder S Rumrill PD. Post-traumatic growth and trauma-informed care in vocational rehabilitation through the lens of the conservation of resources theory. Work. (2023) 75:3–10. doi: 10.3233/WOR-236014, 37092208

[11] Chen S Westman M Hobfoll SE. The commerce and crossover of resources: resource conservation in the service of resilience. Stress Health. (2015) 31:95–105. doi: 10.1002/smi.2574, 25873421 PMC4564014

[12] Shin H Park C. Mastery is central: an examination of complex interrelationships between physical health, stress and adaptive cognition, and social connection with depression and anxiety symptoms. Front Psych. (2024) 15:1401142. doi: 10.3389/fpsyt.2024.1401142, 38751422 PMC11094708

[13] Garcia JM Sirard JR Deutsch NL Weltman A. The influence of friends and psychosocial factors on physical activity and screen time behavior in adolescents: a mixed-methods analysis. J Behav Med. (2016) 39:610–23. doi: 10.1007/s10865-016-9738-6, 27055818

[14] Ahl RE Cook E McAuliffe K. Having less means wanting more: children hold an intuitive economic theory of diminishing marginal utility. Cognition. (2023) 234:105367. doi: 10.1016/j.cognition.2023.105367, 36680975

[15] Matjasko JL Cawley JH Baker-Goering MM Yokum DV. Applying behavioral economics to public health policy: illustrative examples and promising directions. Am J Prev Med. (2016) 50:S13–9. doi: 10.1016/j.amepre.2016.02.007, 27102853 PMC4871624

[16] Onnela JP Rauch SL. Harnessing smartphone-based digital phenotyping to enhance behavioral and mental health. Neuropsychopharmacology. (2016) 41:1691–6. doi: 10.1038/npp.2016.7, 26818126 PMC4869063

[17] Mekary RA Willett WC Hu FB Ding EL. Isotemporal substitution paradigm for physical activity epidemiology and weight change. Am J Epidemiol. (2009) 170:519–27. doi: 10.1093/aje/kwp163, 19584129 PMC2733862

[18] Geraci M Bottai M. Quantile regression for longitudinal data using the asymmetric Laplace distribution. Biostatistics. (2007) 8:140–54. doi: 10.1093/biostatistics/kxj039, 16636139

[19] Shiffman S Stone AA Hufford MR. Ecological momentary assessment. Annu Rev Clin Psychol. (2008) 4:1–32. doi: 10.1146/annurev.clinpsy.3.022806.091415, 18509902

[20] Christensen MA Bettencourt L Kaye L Moturu ST Nguyen KT Olgin JE . Direct measurements of smartphone screen-time: relationships with demographics and sleep. PLoS One. (2016) 11:e0165331. doi: 10.1371/journal.pone.0165331, 27829040 PMC5102460

[21] Sasaki JE John D Freedson PS. Validation and comparison of ActiGraph activity monitors. J Sci Med Sport. (2011) 14:411–6. doi: 10.1016/j.jsams.2011.04.003, 21616714

[22] Kroenke K Strine TW Spitzer RL Williams JB Berry JT Mokdad AH. The PHQ-8 as a measure of current depression in the general population. J Affect Disord. (2009) 114:163–73. doi: 10.1016/j.jad.2008.06.026, 18752852

[23] Steele-Rosomoff R. Pain:clinical manual for nursing practice. Pain. (1989) 38:356. doi: 10.1016/0304-3959(89)90226-1

[24] DeSousa M Reeve CL Peterman AH. Development and initial validation of the perceived scarcity scale. Stress Health. (2020) 36:131–46. doi: 10.1002/smi.2908, 31692256

[25] Sterne JA White IR Carlin JB Spratt M Royston P Kenward MG . Multiple imputation for missing data in epidemiological and clinical research: potential and pitfalls. BMJ. (2009) 338:b2393. doi: 10.1136/bmj.b2393, 19564179 PMC2714692

[26] Jang H Choi HL Kim J. Unmet healthcare needs, gender, and depressive symptoms: evidence from asymmetric fixed effects models. Public Health. (2025) 249:105991. doi: 10.1016/j.puhe.2025.105991, 41092560

[27] Guo H Man M Hu Z Liu Y Chen W Wang S . Screen time, physical activity, sleep, and depression risk in adolescents: an observational study based on the compositional isotemporal substitution model. Front Public Health. (2026) 14:1782512. doi: 10.3389/fpubh.2026.1782512, 42158198 PMC13182083

[28] Wang C Li Y. Heterogeneous and nonlinear associations between depressive symptoms and functional disability in older Chinese adults. BMC Psychol. (2026). doi: 10.1186/s40359-026-04984-7, 42271459 PMC13480101

[29] Deng Y Chang C Ido MS Long Q. Multiple imputation for general missing data patterns in the presence of high-dimensional data. Sci Rep. (2016) 6:21689. doi: 10.1038/srep21689, 26868061 PMC4751511

[30] Grgic J Dumuid D Bengoechea EG Shrestha N Bauman A Olds T . Health outcomes associated with reallocations of time between sleep, sedentary behaviour, and physical activity: a systematic scoping review of isotemporal substitution studies. Int J Behav Nutr Phys Act. (2018) 15:69. doi: 10.1186/s12966-018-0691-3, 30001713 PMC6043964

[31] Shang A Bao H. Self-esteem and appearance anxiety among Chinese college students: the roles of social media use and upward social comparison. Front Psychol. (2025) 16:1562711. doi: 10.3389/fpsyg.2025.1562711, 40528848 PMC12171134

[32] Exelmans L Van den Bulck J. Bedtime mobile phone use and sleep in adults. Soc Sci Med. (2016) 148:93–101. doi: 10.1016/j.socscimed.2015.11.037, 26688552

[33] Schuch FB Morres ID Ekkekakis P Rosenbaum S Stubbs B. A critical review of exercise as a treatment for clinically depressed adults: time to get pragmatic. Acta Neuropsychiatr. (2017) 29:65–71. doi: 10.1017/neu.2016.21, 27145824

[34] Kandola A Ashdown-Franks G Hendrikse J Sabiston CM Stubbs B. Physical activity and depression: towards understanding the antidepressant mechanisms of physical activity. Neurosci Biobehav Rev. (2019) 107:525–39. doi: 10.1016/j.neubiorev.2019.09.040, 31586447

[35] Gjata NN Ullman TD Spelke ES Liu S. What could go wrong: adults and children calibrate predictions and explanations of others' actions based on relative reward and danger. Cogn Sci. (2022) 46:e13163. doi: 10.1111/cogs.13163, 35738555 PMC9284802

[36] Lopes LS Valentini JP Monteiro TH Costacurta MCF Soares LON Telfar-Barnard L . Problematic social media use and its relationship with depression or anxiety: a systematic review. Cyberpsychol Behav Soc Netw. (2022) 25:691–702. doi: 10.1089/cyber.2021.0300, 36219756

[37] Schuch FB Vancampfort D Richards J Rosenbaum S Ward PB Stubbs B. Exercise as a treatment for depression: a meta-analysis adjusting for publication bias. J Psychiatr Res. (2016) 77:42–51. doi: 10.1016/j.jpsychires.2016.02.023, 26978184

[38] Firth J Torous J Stubbs B Firth JA Steiner GZ Smith L . The "online brain": how the internet may be changing our cognition. World Psychiatry. (2019) 18:119–29. doi: 10.1002/wps.20617, 31059635 PMC6502424

[39] Bernstein EE McNally RJ. Acute aerobic exercise helps overcome emotion regulation deficits. Cogn Emot. (2017) 31:834–43. doi: 10.1080/02699931.2016.1168284, 27043051

[40] Abid M Cherni Y Batcho CS Traverse E Lavoie MD Mercier C. Facilitators and barriers to participation in physical activities in children and adolescents living with cerebral palsy: a scoping review. Disabil Rehabil. (2023) 45:4322–37. doi: 10.1080/09638288.2022.2150327, 36447398

[41] Terry J Meara R. A scoping review of deaf awareness programs in health professional education. PLOS Glob Public Health. (2024) 4:e0002818. doi: 10.1371/journal.pgph.0002818, 39159205 PMC11332937

[42] Mansfield M Thacker M Spahr N Smith T. Factors associated with physical activity participation in adults with chronic cervical spine pain: a systematic review. Physiotherapy. (2018) 104:54–60. doi: 10.1016/j.physio.2017.01.004, 28822600

[43] Dai L Su B Liu Q. Influence of grit on adolescents' exercise adherence: the mediating role of exercise self-efficacy and the moderating role of self-control. Acta Psychol. (2025) 255:104952. doi: 10.1016/j.actpsy.2025.104952, 40158229

[44] Dunton GF. Ecological momentary assessment in physical activity research. Exerc Sport Sci Rev. (2017) 45:48–54. doi: 10.1249/JES.0000000000000092, 27741022 PMC5161656

[45] Ayele DG Abdallah ASR Mohammed MOM. Determinants of under-five children body mass index in Sudan; application of quantile regression: a systematic review. Iran J Public Health. (2021) 50:1–10. doi: 10.18502/ijph.v50i1.5067, 34178759 PMC8213622

[46] Okely AD Kontsevaya A Ng J Abdeta C. 2020 WHO guidelines on physical activity and sedentary behavior. Sports Med Health Sci. (2021) 3:115–8. doi: 10.1016/j.smhs.2021.05.001, 35782159 PMC9219310

[47] Martin Ginis KA van der Scheer JW Latimer-Cheung AE Barrow A Bourne C Carruthers P . Evidence-based scientific exercise guidelines for adults with spinal cord injury: an update and a new guideline. Spinal Cord. (2018) 56:308–21. doi: 10.1038/s41393-017-0017-3, 29070812

[48] Bal VH Wilkinson E Glascock V Hastings RP Jahoda A. Mechanisms of change in behavioral activation: adapting depression treatment for autistic people. Cogn Behav Pract. (2023) 30:589–96. doi: 10.1016/j.cbpra.2022.03.006, 37899797 PMC10611425

[49] Heath GW Levine D. Physical activity and public health among people with disabilities: research gaps and recommendations. Int J Environ Res Public Health. (2022) 19:10436. doi: 10.3390/ijerph191610436, 36012074 PMC9408065

[50] Appelbaum PS. Protecting the rights of persons with disabilities: an international convention and its problems. Psychiatr Serv. (2016) 67:366–8. doi: 10.1176/appi.ps.201600050, 27032795

[51] Nahum-Shani I Smith SN Spring BJ Collins LM Witkiewitz K Tewari A . Just-in-time adaptive interventions (JITAIs) in Mobile health: key components and design principles for ongoing health behavior support. Ann Behav Med. (2018) 52:446–62. doi: 10.1007/s12160-016-9830-8, 27663578 PMC5364076

